# The efficacy of trazodone in reducing stress related behaviours in hospitalised dogs or dogs confined postsurgery

**DOI:** 10.18849/ve.v8i2.550

**Published:** 2023-05-26

**Authors:** Lara Dillon+

**Keywords:** DOGS, STRESS, TRAZODONE, POSTSURGICAL CONFINEMENT, VETERINARY PRACTICE, ANXIETY, ANXIOLYTIC

## Abstract

**PICO question:**

In hospitalised dogs or dogs confined postsurgery, does administration of trazodone reduce stress related behaviours compared to no treatment with trazodone?

**Clinical bottom line:**

**Category of research:**

Treatment.

**Number and type of study designs reviewed:**

Three papers were critically reviewed. One was a prospective, randomised, blinded observational study, another was a randomised, placebo-controlled clinical trial, and the last was a non-randomised prospective, open-label clinical trial.

**Strength of evidence:**

Weak.

**Outcomes reported:**

The administration of trazodone to hospitalised dogs reduced several observed stress related behaviours compared to a control group that was environmentally matched to the treatment group (Gilbert-Gregory et al., 2016). In dogs subjected to postsurgical confinement at home, trazodone administration was not more effective at reducing stress related behaviours compared with a placebo in one study (Gruen et al., 2017); however, it was effective when observed in a non-placebo controlled clinical trial (Gruen et al., 2014). Further investigation with a larger sample size would assist in strengthening the evidence of an association between trazodone administration and a reduction in the behavioural signs of stress in dogs.

**Conclusion:**

The available evidence weakly supports the hypothesis that administration of trazodone is an effective treatment in reducing stress related behaviours in hospitalised dogs and dogs confined post-surgery, and further studies are required to confirm its efficacy. The quality of the evidence when hospitalised dogs was studied was moderate (Gilbert-Gregory et al., 2016), however in dogs studied that were confined postsurgery, the evidence is weaker (Gruen et al., 2014; Gruen et al., 2017). Different trazodone doses were evaluated in the studies and so further studies focusing on dose effects are required to determine appropriate dose rates. Further studies also need to be conducted to evaluate the appropriate length of time that trazodone should be given prior to a stressful event, as well as whether trazodone needs to be used in conjunction with other anxiolytic drugs to optimise efficacy.

## How to apply this evidence in practice

The application of evidence into practice should take into account multiple factors, not limited to: individual clinical expertise, patient’s circumstances and owners’ values, country, location or clinic where you work, the individual case in front of you, the availability of therapies and resources.

Knowledge Summaries are a resource to help reinforce or inform decision making. They do not override the responsibility or judgement of the practitioner to do what is best for the animal in their care.

## Clinical scenario

You are a small animal veterinarian working in a private practice with a high surgical case-load. The recognition and reduction of iatrogenic stress has become a large focus in companion animal veterinary practice, especially with the increase in patients undergoing longer hospitalisation stays to receive postoperative care. Trazodone, an anxiolytic, is increasingly used in dogs to reduce stress and stress related behaviours. However, there have been debates between colleagues about its efficacy. An example is a 5-month-old puppy that was hospitalised after a castration procedure. In recovery the puppy became quite stressed – vocalising and trying to dig out of its cage – and your colleague recommended giving trazodone. You decide to investigate the efficacy of trazodone in hospitalised patients and those confined postsurgery.

## The evidence

Three studies evaluated the efficacy of trazodone in reducing stress related behaviours in dogs undergoing hospitalisation, or postsurgery confinement. One prospective, randomised, blinded observational study compared the behaviour observed after trazodone administration with no trazodone administration in environmentally matched dogs that were hospitalised after surgery (Gilbert-Gregory et al., 2016). Through the use of a blinded observer, randomisation, and matching the environment of the treatment and control dogs, the quality of evidence in this study is moderate. Another randomised, placebo-controlled clinical trial compared the stress related behaviours of two groups of dogs who were confined after orthopaedic surgery, who were administered either trazodone, or a placebo (Gruen et al., 2017). Use of a placebo strengthens the evidence in this study, however utilising a small number of dogs for the whole study (n = 29) makes it weak. A third prospective, non-randomised, open-label clinical trial evaluated the behaviour, compared to baseline behaviours, of dogs administered trazodone after orthopaedic surgery and subjected to postsurgery confinement and rest (Gruen et al., 2014). However, the strength of the evidence in this study is reduced due to a lack of comparison with a placebo or control group, and a small study size (n = 36), deeming the evidence weak. Overall, the available evidence is weak.

## Summary of the evidence

**Gilbert-Gregory et al. (2016):** 

Population:	Recruitment: Client-owned dogs admitted to Ohio State University Veterinary Medical Center for a non-specified surgical procedure from June 22 to October 8, 2015. Collection of history and a physical examination were performed prior to enrollment. Criteria for eligibility and inclusion for treatment group: Dogs prescribed trazodone hydrochloride at any point during hospitalisation. Criteria for eligibility and inclusion for control group: Dogs not administered trazodone during hospitalisation. Selected on the basis of similar housing environment and proximity within the hospital to the treatment dogs to the control dogs for environmental variables that could be associated with stress. Criteria for exclusion and rejection from both groups: If they had been administered oral or injectable medications that could alter mentation ≤ 2 hours before observation.
Sample size:	120 dogs.
Intervention details:	Group allocation: There were two groups: Treatment group (received trazodone) (n = 59 dogs) and control group (did not receive anything) (n = 58 dogs). One dog in the control group was matched to a dog in the treatment group in a similar housing environment. The age, body weight, sex, ratio of mixed-breed to purebred dogs, hospital admission service, time interval between the two behavioural observations, and the number and proportion of dogs that were concurrently receiving mentation-altering medications (besides trazodone) were not significantly different between the treatment and control groups. Procedure: Trazodone was administered to dogs displaying signs of fear, anxiety or aggression (unless contra-indicated for the patient), and these dogs formed the treatment group. Trazodone was administered at a starting dose of 3.5 mg/kg every 12 hours by a veterinary student or a veterinary assistant / technician hidden inside food. Each dog was monitored to ensure that the medication was consumed. The medication was not administered to patients who were not being given food at the time; i.e., prior to surgery, or who were not being fed orally, such as through a feeding tube. Some dogs who had been in surgery were concurrently administered tramadol hydrochloride at the discretion of the clinical veterinarian. Treatment was continued throughout the hospitalisation period as needed based on stress related behaviour. Adjustments to dosage were made at the discretion of the attending clinician as needed.
Study design:	Prospective, randomised, blinded controlled study.
Outcome studied:	17 signs and / or behaviours associated with fear, anxiety and aggression grouped into three behavioural summation categories that were observed at time 1 (≤ 45 minutes after trazodone administration) and at time 2 approximately 90 minutes after the first observation. These behaviours were: Frenetic: lip licking, pacing, panting, spinning, trembling, whining, yawning, wet dog shake, pupil dilation, lifting a forelimb. Fractious: growling, lunging, showing teeth, pupil dilation, lifting a forelimb. Freeze: averting gaze, pinning back ears, whale eye sign, pupil dilation, lifting a forelimb. The number of observed stress related signs or behaviours were summed for each group, with each dog receiving a total stress related score and a behaviour summation score that was compared between time 1 and time 2. Behavioural assessment: Once paired dogs were selected, a blinded observer performed 60 second behavioural observations on each of the dogs, observing them for 30 seconds from a distance of 3 feet, and then for an additional 30 seconds directly in front of the door. The dogs were assessed one after another, and the order was maintained when the next observation occurred. The first observation was conducted ≤ 45 minutes after trazodone administration to the treatment dog. If the first observation was performed more than 45 minutes after administration of trazodone, the dog was removed from the study for undisclosed reasons. The second observation was conducted approximately 90 minutes after the first observation. Observations were conducted utilising a checklist developed by the first author in conjunction with a board-certified veterinary behavioural specialist.
Main Findings (relevant to PICO question)	117 dogs from an original sample size of 120 dogs: One dog was removed from the treatment group as the first observation was performed more than 45 minutes after trazodone administration, and two dogs from the control group were removed as they were not present in the ward when the second round of observations were due to occur. Treatment with trazodone resulted in a reduction in the observed signs of stress and stress related behaviours. This observation was made when a sign from time 1 was absent when observed during time 2. In the treatment group this included a 29% reduction in lip licking (P = 0.007), 34% reduction in panting (P < 0.001), 24% reduction in whining (P = 0.004), and 24% reduction in whale eye sign (P = 0.004). There was no significant reduction in the other observed signs. The control group did not show a reduction in stress related signs, besides a 24% reduction in whale eye sign (P = 0.031). Median total stress related behaviour scores, and median summation scores for frenetic and freeze behaviours were lower at time 2 compared to time 1 for the treatment group (P ≤ 0.009), while they were not significantly lower for the control group. The fractious behaviours were not reduced between time 1 and time 2 for the treatment or control group.
Limitations:	During the study, 54% (32/59) of the dogs that received trazodone were also administered one or more concomitant medications. Most (18/32) received non-steroidal anti-inflammatories (NSAIDs) (carprofen, deracoxib, tepoxalin, firocoxib or meloxicam) or tramadol (14/32), however some (9/32) were also administered gastroprotectants (omeprazole, sucralfate, pantoprazole sodium, or famotidine), systemic antimicrobials (6/32) (metronidazole, cephalexin, amoxicillin-clavulanic acid, enrofloxacin, ampicillin sodium-sulbactam, marbofloxacin, or amikacin sulfate), opioids (6/32) (hydromorphone hydrochloride, butorphanol tartrate, fentanyl citrate, or methadone hydrochloride), a tranquilliser (5/32) (acepromazine maleate), an anti-epileptic with analgesia (4/32) (gabapentin), a corticosteroid (4/32) (prednisolone), antiemetics (4/32) (ondansetron hydrochloride, maropitant citrate, or metoclopramide hydrochloride), an antihistamine (3/32) (diphenhydramine hydrochloride), an appetite stimulant (1/32) (mirtazapine), a laxative (1/32) (lactulose), and an anthelmintic (1/32) (fenbendazole). Any or all of these could have contributed to a reduction of stress related behaviour in the dogs. There is also the potential for drug interactions to occur, especially those that induce the cytochrome p450 system. There was potential for selection bias to have impacted which dogs were chosen for either treatment or control group based on their initial behaviour as this was not randomised and treatment dogs were chosen on the basis of their perceived level of stress in hospital. The environmentally matched dogs were not true controls as they may not have had the same levels of stress at baseline – this was not tested and accounted for as the dogs’ baseline behaviour was not observed. The behaviours were only analysed within groups and not between groups whereby the same behaviours were not observed by both the treatment and control group at time 1. There was a higher chance of type I errors (false positives for trazodone efficacy) when multiple comparisons were made between and within groups without adjusting the P value. Whether the dog had undergone surgery or not, the time in recovery if surgery had occurred, or duration of the hospital stay overall was not taken into consideration when selecting the dogs for the control group, and was in fact significantly different (P = 0.015) between treatment and control groups. When comparing the time of assessment pre or post surgery, there were a higher proportion of dogs evaluated who had surgery pending in the treatment group than surgical patients who were recovering post surgery, compared to the control group where there was an equal number of dogs evaluated either pre or post surgery, potentially exacerbating the stress related behaviours in time 1, and the changes between when they were first evaluated compared to the second evaluation. There was a higher proportion of postoperative patients in the control group compared to the treatment group, therefore the changes between time 1 and time 2 may have been less obvious compared to the treatment group as other factors such as the anaesthetic, pain and duration of hospitalisation may all have contributed to stress related behaviours or lack thereof. When selecting the environmentally matched dogs, duration of hospitalisation was not considered. It is therefore possible that the lack of changes of behaviour between time 1 and time 2 may have been due to the dogs having already been in hospital for longer compared to the treatment dogs, and had already acclimatised to the environment.

**Gruen et al. (2017):** 

Population:	Recruitment: Dogs that were admitted to the North Carolina State Veterinary Hospital Orthopedic Surgery Service. Patients recruited were enrolled in the study if they met the criteria for eligibility and exclusion following physical examination and collection of patient history, including history of prescription of mentation-altering drugs. As an incentive for participation, enrolled participants were provided with free postoperative laboratory tests and $100 off the price of postoperative radiographs 8 weeks after surgery. Criteria for eligibility and inclusion: Owned, with attainment of informed owner consent.
Sample size:	33 dogs.
Intervention details:	Group allocation: Control group (n = 15 dogs) administered placebo and treatment group (n = 14 dogs) administered trazodone. There were no differences between the distribution of sexes, mean age or mean weight between the treatment and placebo groups. The enrolled dogs were undergoing either stifle stabilisation (n = 23), fracture repair or external fixator placement (n = 5), or a total hip replacement (n = 1). There was no significant difference (Fisher exact test, p = 1.00) between the groups in the distribution of types of surgery by location Procedure: Owners of enrolled dogs completed a presurgical questionnaire about the dogs’ baseline behaviour to determine; baseline activity level, degree of control of dog during leashed walks, tolerance of confinement. Cases were assigned randomly to either control or treatment group. Owners of enrolled dogs, veterinarians, technicians and members of staff were blinded to the treatment except when needed. After surgery owners were given standard postoperative instructions for confinement and reducing activity for 4 weeks. During this time period, owners were to administer: days 1–10, daily nonsteroidal anti-inflammatory; days 1–7, oral tramadol (4–6 mg/kg every 12 hours); and days 1–7, half dose of treatment (either trazodone at 3–5 mg/kg every 12 hours, or an equivalent tablet size and quantity of placebo given every 12 hours). When tramadol was discontinued after day 7, the dose of the treatment was increased to the assigned level (trazodone at 5–7 mg/kg every 12 hours or an equivalent tablet size and quantity of placebo given every 12 hours) and continued for 4 weeks. Dosage was altered without releasing the blinding if any of the following occurred: The dose of treatment seemed too high for a dog (e.g., if the owner reported that the dog was excessively sleepy) and could be decreased. The owner reported lack of efficacy, and was given an increased dose. The owner reported lack of efficacy and wanted to withdraw – the assigned treatment would cease and known trazodone would be dispensed for the duration of the study. Each week, owners were required to submit an emailed questionnaire about their dog’s behaviour, with a follow-up call if they did not return the questionnaire on time. Questions included: Tolerance of confinement and restriction of activity. Control of the dog on leash walks. Overall activity levels. Confirmation of dosing schedule. Reporting of any adverse events related to medication. Owner satisfaction with the treatment (either placebo or trazodone). Owners were called after 4 weeks, whereby they could elect to continue administration of treatment for up to 8 weeks postoperatively. The final dosage range received ranged from 5.6–21.6 mg/kg/day. 25 of the 29 dogs that were enrolled in the study completed it, with 3 in the trazodone group and 1 in the placebo group withdrawing. At the 4 week time point, most owners in both the trazodone (11/11 [100%]) and placebo (12/14 [85.7%]) groups elected to continue the assigned treatment at the assigned dose.
Study design:	Randomised placebo-controlled clinical trial.
Outcome studied:	The behaviours studied related to the dog’s behaviour postsurgery with treatment administration, and were compared to baseline behaviours determined before surgery: Greeting behaviour. Calmness. Willingness to be controlled on leash. Pulling. Tolerance of confinement at home. Reported by owners, based on questionnaires.
Main Findings (relevant to PICO question)	29 dogs from an original sample size of 33 dogs: Dogs were removed from the study when diagnosed with a clinical disease during the study (n = 1), the owner did not return the questionnaire or the owner removed the dog from the study due to lack of efficacy of treatment (n = 3). In baseline ratings, no significant differences were found between the trazodone and placebo groups for greeting behaviour (P = 0.21), calmness (P = 0.15), willingness to be controlled on leash (P = 0.86), pulling whilst on leash (P = 0.53), or tolerance of confinement at home (P = 0.68). When baseline and post-treatment ratings were compared, there were no significant differences found between the trazodone and placebo groups for greeting behaviour (P = 0.69), calmness (P = 0.39), ability to be controlled on leash (P = 0.69), pulling whilst on leash (P = 0.12), or tolerance of confinement at home (P = 0.68). The treatment was rated as moderately or extremely helpful in 8/11 (72.7%) owners of the trazodone group and 11/14 (78.6%) of owners of the placebo group for facilitating both calmness and manageability. There were no significant differences between groups in regard to the duration of the effect (P = 0.84), or the change in the effect over time (P = 0.62). Therefore, when compared with a placebo, trazodone did not demonstrate an effect on calmness, manageability and stress reduction that was more effective than the placebo.
Limitations:	Systemic bias may have existed due to the existing use of trazodone at the clinic for postsurgical confinement, as veterinary surgeons at the clinic were already convinced about the efficacy of trazodone, and therefore were hesitant to enrol their own patients in the trial in case they received a placebo, contributing to a reduced number of study participants. Case selection when enrolling participants may have been biased towards dogs that were inherently calm and more accepting of confinement to reduce the potential for complications postsurgery due to the preconceived efficacy of trazodone. There was no evidence or information provided about why particular parameters were chosen to assess behaviour, and the investigators did not justify their selection of behaviour markers of calmness, manageability and stress. Owners’ evaluation is subjective and may not have been entirely accurate, with the potential for social desirability bias on the part of the owners have an effect. Owners may not have properly followed prescribed medication and activity regime. Placebo-by-proxy effect may have affected results, whereby the belief of the owners that their dog is receiving an anxiolytic medication could change the way that the owner interacts with the dog and could thereby lead to an actual change in the dog's behaviour due to the interaction. The outcome assessment tool used may not have been sensitive enough to capture the differences between the groups. Sample size determined by a pilot study (Gruen et al., 2014) was not large enough (Dhand & Khatkar, 2014) to capture the effects when utilised in a placebo controlled study.

**Gruen et al. (2014):** 

Population:	Recruitment: 36 client-owned dogs that underwent orthopaedic surgery at North Carolina State University College of Veterinary Medicine Veterinary Health Complex. Criteria for eligibility and inclusion: In good health and weighed at least 5 kg (11 lb). Owned, with attainment of informed owner consent. Not receiving concomitant behavioral medications or monoamine oxidase inhibitors, such as amitraz products before the study commenced. Castrated males and spayed females. Owners agreed to administer medication as required, report adverse effects if they occur, and complete weekly online surveys describing features of their dog’s behaviour.
Sample size:	41 dogs: 25/41 (52.2%) were female, the mean age was 3.0 ± 2.46 years, and the mean weight was 32.0 ± 10.6 kg (70.4 ± 23.3 lb). Study participants underwent a variety of orthopaedic surgeries, and were grouped into surgery type by hip joint (n = 11 dogs), stifle joint (n = 21 dogs), and other (n = 4 dogs).
Intervention details:	All dogs were administered trazodone, with the initial dose being 5 mg/kg, PO, q 12 h before moving to the standard dose of approximately 7 mg/kg, PO, q 12 h. The standard dosage was determined by the authors in accordance with a small pilot study (Gruen, 2012). After week 2, the owners of 25 dogs requested a higher dosage of trazodone, and these dogs became the high-dosage group (mean total daily [within 24 hr period] peak dosage was 21.19 ± 39 mg/kg, PO). 11 dogs received the standard dosage (mean, 6.86 mg/kg, q 12 h). Procedure: When enrolled, the owners completed a presurgical survey evaluating their dog’s behaviour. Surveys asked owners to rate the following on a scale from 1–4 with lower scores indicating calmness and manageability: Tolerance of confinement when left alone. Tolerance of confinement when owner is home. Tendency to pull on a leash during walks. Willingness to be controlled by the owner. Intensity of greeting behaviour to the owner and other familiar people. Overall calmness. Owners were sent questionnaire (via email) and asked to complete the same online survey evaluating their dog’s behaviour each week for 4 weeks. A fifth survey was completed 8–12 weeks after surgery at the patient’s final postoperative evaluation. For days 1–3 postsurgery, owners were instructed to administer trazodone at half the standard dose (approximately 3.5 mg/kg every 12 h) and tramadol for pain management (4–6 mg/kg every 8–12 h) – the researcher’s rationale for this was to establish initial blood concentrations of trazodone, reduce the possibility of receiving excess serotonin, and to develop tolerance to the fluctuating sedative effect of trazodone (the authors did not disclose why the dose was approximate, however it is assumed that it was due to the tablet size and dose of trazodone per tablet in milligrams). After 3 days, tramadol administration ceased, and trazodone dosage was increased to the standard dosage (approximately 7 mg/kg, every 12 h). During the study, if the standard dosage was considered insufficient by the owner, the dosage and administration schedule of trazodone were increased after consultation with a veterinary behaviourist (approx 7–10 mg/kg, every 8 h). Total daily dose was calculated as the total amount of trazodone administered PO during a 24 hour period. Administration of trazodone continued for 4 weeks postoperation.
Study design:	Prospective non-randomised open-label clinical trial.
Outcome studied:	The effect of trazodone on tolerance to confinement and calming by comparing baseline behaviours before surgery, and behaviour after surgery with trazodone administration: Intensity of greeting behaviour to the owner and other familiar persons. Overall calmness. Willingness to be controlled by the owner. Tendency to pull on a leash during walks. Trazodone dosage. Trazodone onset of action and duration. Adverse events after trazodone administration.
Main Findings (relevant to PICO question)	36 dogs from an original sample size of 41 dogs: Dogs were withdrawn from the trial when the owner was not compliant with online surveys and communication (n = 4), and / or it was determined that they were concurrently receiving mentation-altering medications (n = 1 dog). In the final survey 8–12 weeks after surgery (depending on the surgery) 32/36 (89%) of dogs improved in their calmness and tolerance to confinement either moderately or extremely. When compared with the presurgical surgery responses, the dogs significantly improved with respect to the intensity of greeting behaviour to the owner and to other familiar persons (P = 0.003) and in overall calmness (P = 0.032). The dogs did not improve with respect to willingness to be controlled by the owner (P = 0.492) and tendency to pull on a leash during walks (P = 0.097). No owners rated trazodone as not at all helpful with regard to facilitating confinement tolerance. In some final surveys, trazodone was reported as extremely helpful in dogs that initially resisted confinement, compared with those that initially accepted confinement (P = 0.011). An exact number of dogs that initially resisted confinement versus dogs that initially accepted confinement was not disclosed. Approximately 90% (37/41) of owners reported that trazodone was moderately or extremely efficacious in positively effecting the dogs confinement and calmness. The mean standard dose (low dose group, n = 11) over 24 hours was 13.73 mg/kg and mean peak dosage over 24 hours (high dose group, n = 25) was 21.19 ± 6.39 mg/kg, PO. There was no significant difference between the two groups in regards to age (P = 0.568), weight (P = 0.770), or surgery type (P = 0.703). The median time for trazodone to cause an effect was 31–45 minutes after administration, with 37/41 (90%) of owners reporting effect between 16 and 90 minutes post administration. The median duration of action of trazodone was ≥ 4 hours. Twenty dogs reported adverse events including: soft stool, loose stool or diarrhoea (1), constipation (1), increased thirst (1), signs of anxiety, restlessness, or agitation (2), aggression (1), moaning (1), drowsiness (2), somnolence (5), panting (2), teeth chattering (1), drooling (1), signs of paranoia (1), incontinence (1). Two instances occurred where dogs accidentally received higher doses than prescribed: one received total dose of 600 mg (20 mg/kg) and was slightly sleepy; the other was given tramadol longer than prescribed concurrently with the standard trazodone dose and no adverse event was reported.
Limitations:	Any dog could receive other concomitant drugs including NSAIDs, buprenorphine, amantadine, or antimicrobials during the study period depending on the veterinary surgeons prescription, which may have impacted on their behaviour. Three dogs received fluoxetine or acepromazine, however only one dog was withdrawn from the study for receiving a drug that may have altered its behaviour. Two dogs received gabapentin for pain which may have influenced stress related behaviour due to its anxiolytic and / or analgesic properties (Papich, 2016a). The study design did not include a control group. It was not stated whether there was a difference in behaviour after administration between the dogs who received a higher dose of trazodone versus a lower dose. Positive effects may have also been due to the dog acclimatising to confinement. A change in behaviour may also have been due to behavioural correction by the owner. A placebo-by-proxy effect may have influenced the dog’s behaviour, whereby the owners may have acted differently around the dogs due to the knowledge of trazodone administration and effects of surgery and elicited a calm response in return. Results depended on the owner’s accurate evaluation of the dog’s behaviour, and the compliance with confinement and calmness parameters. Results depended on owners’ compliance in treatment administration.

## Appraisal, application and reflection

### Behaviour as a measure of stress

In all three papers, the primary outcome measured was the reduction in stress related behaviours (Gilbert-Gregory et al., 2016), and increased calmness and manageability (Gruen et al., 2014; and Gruen et al., 2017). Gilbert-Gregory et al. (2016) assessed this through creating a checklist comprised of 17 signs and behaviours that were associated with fear, anxiety and aggression. Gruen et al. (2014) assessed intensity of greeting behaviour, overall calmness, willingness to be controlled and pulling on the leash, and Gruen et al. (2017) similarly assessed stress related behaviour in five provocative situations (tolerance of confinement when left by themselves, tolerance of confinement when the owner was home, tendency to pull on the leash whilst on walks, willingness to be controlled by the owner such as through obeying familiar commands, and intensity of greeting behaviour towards the owner and other known persons), and one temperament measure (overall calmness). Provided correct behaviours are observed and observers are well trained and accustomed to using these tools, assessing specific postures, mannerisms and activities related to stress can be an accurate measurement tool (Beerda et al., 1997; Beerda et al., 1998; and Overall, 2013). However, it is important to consider that dogs who are not inherently stressed normally may not demonstrate a change in behaviour after trazodone administration, and a large sample size would be needed to accommodate for this. There is also the requirement to determine the difference between pain related behaviour and stress related behaviour, as the two often overlap and different pain management strategies and type of procedure can impact the amount of stress related to this, as well as individual pain tolerance thresholds.

An animals’ behaviour when it is experiencing stress depends on numerous factors such as genetic predisposition, past experiences, and the current environment (Lloyd, 2017)**. **Thus age, sex, health status, and any previous experiences they may have had during hospitalisation or confinement all of which may impact stress related behaviour and therefore may inflate or reduce the perceived effect of trazodone. The type of behaviour elicited by a dog when it is experiencing stress depends on the type of threat that it is perceiving, whether it is escapable or inescapable, as well as personal characteristics including individual variations in coping styles (Lloyd, 2017; and Steimer, 2022).

Gilbert-Gregory et al. (2016) acknowledges the variety of behaviours displayed when a dog is experiencing stress, and draws from physiologic knowledge through the creation of the behavioural checklist utilised in the study, however Gruen et al. (2014; and 2017) does not describe why the factors in their checklist were chosen, or how they reflect increased manageability and calmness, or a reduction in stress. This makes it more difficult to interpret the results, and compare the findings to other studies.

### Canine specific stress related behaviours

Responses to stressful stimuli can vary between individual species, therefore it is important to analyse and interpret the behaviour of dogs through referring to species-specific behaviours and responses. Studies such as those by Beerda et al. (1997; and 1998) aimed to determine the behavioural, hormonal and physical responses elicited by stressful and provocative stimuli such as electric shock. The responses typically involved vocalisation, trembling, altered posture usually into a lowered position, averting their gaze and displaying the whites of the eyes in a distinctive ‘whale eye’ sign, along with quickened breathing, lifting a paw, yawning, circling, and generally becoming more agitated, escalating to urination and / or defecation in some instances (Beerda et al, 1997; and Beerda et al., 1998). These behaviours were accompanied by an increased heart rate, along with increased levels of salivary cortisol, indicative of a stress response (Beerda et al., 1997)**. **More subtle signs of stress and anxiety that have been described in dogs include darting eyes and scanning for potential dangers or means of escape, refusing treats or becoming inappetent in general, and appearing distracted by sniffing, sighing or stretching (Overall, 2013).

Gilbert-Gregory et al. (2016) developed the checklist utilised in their study with the assistance of a board-certified veterinary behaviourist. The 17 behaviours utilised in this checklist echo the responses observed in numerous studies (Beerda et al., 1997; and Beerda et al., 1998), strengthening the conclusion that these behaviours were indeed stress related. The first author also mentored the observer for 2 weeks before the commencement of the study on how to properly identify the behaviours through conducting preliminary investigations on all dogs housed at the hospital (n = 100), and comparing scores produced concurrently by both the first author and the observer (Gilbert-Gregory et al., 2016). This increased interobserver reliability, ensured that the observer was aware and skilled in recognising stress related signs and behaviours.

Gruen et al. (2014) developed the survey utilised in the study through evaluating the dog’s responses to situations that were deemed relevant to successful management after orthopaedic surgery, including greeting behaviour and the level of calmness and manageability that the dog demonstrated. However, the authors did not specify why these parameters were chosen to demonstrate tolerance of confinement. The study by Gruen et al. (2017), utilised this assessment tool (Gruen et al., 2014), however, the authors commented that non-specific behavioural changes due to trazodone administration may have been missed if the tool was not sensitive enough to be used in a placebo-controlled trial.

One important consideration that may have been overlooked in these studies is the potential for the involvement of different emotions in these stress related responses. For example, a dog confined at home in a crate may appear stressed, not due to anxiety, but due to being unable to undertake normal behaviours that they are accustomed to such as having free roam of the house, eliciting high arousal, excitement or frustration. A dog confined in a hospital situation may be displaying stress related behaviours due to high energy that cannot be expelled due to confinement, or frustration due to separation from the owner.

### Administration of other behaviour altering drugs as a confounding factor

In the study by Gilbert-Gregory et al. (2016), 32/59 (54%) of the dogs enrolled in the trazodone group were also administered one or more than one concomitant medications that were prescribed by the attending clinicians. Whilst no adverse reactions were reported through the use of more than one drug, these medications included opioids, tranquillisers and gabapentin which are known to alter mentation (Papich, 2016a; Papich, 2016b; and Papich, 2016c) and may therefore impact stress related behaviours. The study did try and account for this as the study participants were excluded if they had been administered oral or injectable mentation altering medications within 2 hours of observation, however there was still the possibility that the results may have been confounded by any administration of these medications with trazodone during their hospitalisation.

Similarly, in Gruen et al. (2014; and 2017), potential mentation-altering drugs were also administered to the patients undergoing observation. A dog that has been administered any of these drugs may display reduced stress related behaviours that are also commonly seen in dogs administered trazodone (Papich, 2016a; Papich, 2016b; Papich, 2016c; and Papich, 2016d), therefore if they were administered within the observation period of the study, they may have been altering the subjects’ behaviour, rather than the actual treatment.

### Pain as a confounding factor

Pain presents in animals in a variety of ways, with specific and non-specific pain behaviours recognisable in dogs (Gaynor & Muir, 2009)**. **Some of these behaviours could be mistaken by an observer as demonstrating a reduction in anxiety. For example, a dog in pain may become hunched up and be reluctant to move as pain progresses and worsens, which could be mistaken for being relaxed, compared to a dog that is lunging and growling (Steimer, 2022)**. **Similarly, pain may be mistaken for anxiety, whereby a dog in pain due to a limb injury may lift its paw and not weight bear on the limb, while an anxious dog may demonstrate a freeze behaviour through lifting a forelimb (Overall, 2013; and Gaynor & Muir, 2009). A dog in pain may also experience stress and demonstrate stress related behaviours due to the pain itself.

Gilbert-Gregory et al. (2016) reduced the effect of pain as a confounder through mentoring the observer for two weeks prior to the commencement of the study, as mentioned previously. This increased the likelihood that what the observer recorded were true and accurate assessments of behavioural signs of stress, rather than confusing them with signs of pain, assuming that the training was sufficient to determine the difference. Certain behavioural observations were also removed from the list that may be confused with pain, such as hunching the hindquarters, reducing the effect of pain on the results (Gilbert-Gregory et al., 2016).

The potential for pain to confound results was evident in the studies by Gruen et al. (2014; and 2017), as the owners were responsible for observing their dogs and reporting their behaviours. It is not specified whether the owners received any training or mentorship regarding how they assessed their dogs. In Gruen et al. (2014), all dogs concurrently received NSAIDs but they did not specify the course length or dose. Therefore, dogs in this study may have appeared more calm and manageable, and less stressed due to increased pain felt as the pain relief medication was removed, or subsequently wore off.

### Hospitalisation as a confounding factor

Whilst the effect of hospitalisation, and any environmental stressors such as fire alarms, building construction, and general foot traffic was reduced in the study by Gilbert-Gregory et al. (2016) through environmentally matching the treatment and control dogs, it is not specified whether the reason for hospitalisation was accounted for when choosing the study subjects. Therefore, some procedures may have impacted more on stress levels compared to others through being more invasive, therefore causing more pain, having longer or shorter surgery time, or through the utilisation of different premedications and anaesthetic drugs which may have provided anxiolysis such as benzodiazepines (Grimm et al., 2015)**.**

The physical effects of being anaesthetised may also have an effect on whether dogs exhibit signs of stress. Dogs that are cold after anaesthesia may shake, and vice versa a dog that is warm and pyrexic due to an illness, or becoming too hot during surgery, may pant (Grimm et al., 2015). It is not discussed how long after surgery dogs were evaluated in the Gilbert-Gregory et al. (2016) study, therefore what may have been perceived as stress induced behaviours, may have instead been due to the physical effects of anaesthesia or surgery. Gilbert-Gregory et al. (2016) reduced the confounding impact of hospitalisation through excluding particular behaviours from the final checklist that were difficult to distinguish as indicators of stress due to the physical limitations or discomfort caused by their various ailments, as mentioned previously. However, there was also a significant (P = 0.015) difference between the groups in regards to surgical status (Gilbert-Gregory et al., 2016), whereby more of the treatment group participants were awaiting surgery, compared to a more even split between patients who had undergone surgery and those that were waiting in the control group. Patients awaiting surgery may have displayed more stress related behaviours compared to those that had already undergone an operation due to the effects previously discussed, which may have affected the results.

In Gruen et al. (2017), there was no significant difference between the trazodone and placebo groups in the distribution of surgery types by the surgical site (P = 1.0). As all dogs received trazodone in Gruen et al. (2014), and were rated by comparing their behaviour before and after trazodone administration, hospitalisation and surgery type were less likely to be confounders. However, the type and duration of postsurgical confinement were not specified in either study (Gruen et al., 2014; and Gruen et al., 2017), therefore calmness and manageability may have been directly affected by the type and duration of confinement.

### Subjective measurement and assessment

Whilst there are studies that evaluate specific behavioural indicators of stress in dogs (Beerda et al., 1997; and Beerda et al., 1998), appraisal of behaviour can differ between operators. As mentioned previously, Gilbert-Gregory et al. (2016) attempted to minimise the impact of interoperator variability through the first author mentoring the observer for a 2 week period prior to the commencement of the study. After this, a preliminary investigation occurred whereby the observer and first author scored all dogs in the study hospital for another 2 weeks in order to evaluate the reliability between the two investigators. The trained observer was blinded to the treatment given, and assessed all study participants (Gilbert-Gregory et al., 2016).

In both studies, Gruen et al. (2014; and 2017) reported a large amount of variability between the reported observations of stress and calmness in dogs as the owners of the subjects were the assessors. Owner’s observations could have been made more reliable through filming the dogs and sending footage to a trained assessor, or providing the owners with detailed instructions as to how to observe and assess their dogs’ behaviour.

### Blinding of assessors and administrators

Blinding is used in clinical trials to remove any bias that can be caused intentionally or unintentionally if participants or the research team are aware of who is receiving an active or placebo treatment (Hadgu et al., 2012). In one of the studies, the observer was blinded as to which dog had received the trazodone treatment, and which dog had not received any treatment (Gilbert-Gregory et al., 2016), and in another the administrators and observers were blinded as to whether their dog received the trazodone treatment or the placebo (Gruen et al., 2017). In the third study, however, all dogs received the trazodone treatment, and no blinding was used (Gruen et al., 2014).

Gruen et al. (2017) discussed the placebo-by-proxy effect and how it may have affected the results through the owners acting differently towards their dogs no matter which treatment they received. This may also have affected the results of the study by Gruen et al. (2014) whereby all dogs received treatment administered by their owners. The authors also highlight the effect that the belief system of the owners clearly had on their ratings of their dog’s behaviour, as three owners with dogs within the treatment group requested known trazodone as they believed that the unknown medication was not benefitting their dogs (Gruen et al., 2017). After the switch, two of these owners then rated trazodone as moderately or extremely effective. One of these dogs had already received an increased dose of trazodone with no improvement, however, when known trazodone was prescribed it was administered at the initial target dosage which was lower than the dose the dog had been receiving before (Gruen et al., 2017). Once the owner was giving the dog what they knew to be trazodone, their ratings of treatment efficacy changed to positive. This could reflect a placebo effect.

### Sample size

When designing a research study, a sample size calculation should always be conducted to determine the number of participants needed to detect a clinically relevant treatment effect (Hammond et al., 2014)**. **If the sample size is too small, a true treatment effect may not be detected (type II error). However, a large sample size may prove unethical (if unnecessary) and costly (Hammond et al., 2014). Gruen et al. (2017) describe their sample size as being based on a priori power calculation utilising the effect from Gruen et al. (2014), whereby 15/17 dogs in the treatment group demonstrated a treatment effect that was deemed to be clinically significant. However, if utilising this expected proportion (88%) from this pilot study, the study by Gruen et al. (2017) would require a sample size of 163 in order to estimate the expected proportion of dogs responding to trazodone with 5% absolute precision and a level of confidence of 95% (Dhand & Khatkar, 2014). The authors also did not provide the justification of their sample size (Gruen et al., 2017).

Gilbert-Gregory et al. (2016) and Gruen et al. (2014), did not specify why certain sample sizes were chosen for the studies and did not report a sample size calculation. In both studies, if an expected proportion of 50% and 95% confidence level were chosen to account for an unknown expected effect of the use of trazodone, then a sample size of 385 in each study would be required to produce a reliable and valid result (Dhand & Khatkar, 2014). However, it would be extremely costly and logistically difficult to recruit that many participants and perform a study of this size.

### Duration of time in a novel environment

Novelty and uncertainty can increase arousal and anxiety by activating the behavioural inhibition system, which inhibits normal behaviours and is one of the first behavioural signs of an anxious state in the dog (Steimer, 2022). When a stimulus is no longer perceived as novel, the anxiety associated with uncertainty is reduced. This is echoed in animals when they acclimatise to their environment and the stressors associated with it. In Gilbert-Gregory et al. (2016), the duration of stay of the dogs was not taken into account when the environmentally matched dogs in the control group were chosen. When analysed, there was a significant (P = 0.01) difference in the mean duration of time in hospital between the treatment and control group dogs, whereby it was shorter for the treatment group (1.5 ± 0.8 days) compared to the control group (2.0 ± 2.0 days) (Gilbert-Gregory et al., 2016). Therefore, the lack of significant decreases in behavioural signs of stress in the control group could reflect acclimatisation to their environment as they had been in the environment for a longer duration of time**. **However, it should be acknowledged that prolonged hospitalisation and time spent in a novel environment may cause increased levels of stress in some dogs**. **In Gruen et al. (2014; and 2017), the impact of residing in a novel environment is next to null as the dogs were evaluated within their own homes, with the main difference from normal potentially being confined to a crate, pen, or particular area of the house compared to free reign throughout the home. Again, it should be acknowledged that this may be a major stressor in some dogs, as well as the stress of imposed restrictions such as reduced exercise.

### Health as a confounding factor

Illness and disease can significantly alter animal behaviour (Frank, 2014). It therefore needs to be considered as a confounding factor when evaluating behaviour as a marker of stress. Only Gruen et al. (2014), specified that dogs must be in good health to be enrolled in the study. Gilbert-Gregory et al. (2016) and Gruen et al. (2017), did not disclose nor specify the health status of the subjects enrolled in their studies, stating that general health checks were conducted, but not disclosing the outcomes of these examinations. As animals were admitted for surgery, they were not disease free.

### Procedural confounding factors

Numerous differences in methodology need to be considered as confounders when evaluating the three studies. In Gilbert-Gregory et al. (2016), a trained veterinarian, veterinary technician or veterinary student administered the treatment to the subjects by hiding it within a treat, reducing confounding factors by maintaining consistency. In comparison, it is unknown how the owners of the subjects in Gruen et al. (2014; 2017) administered the trazodone or placebo, and whether they had been informed of how to give medication appropriately without causing undue stress, and also ensure that the dog actually received the intended dose. This would have increased the possibility of procedural confounding factors having an effect on the results.

Other potential procedural confounding factors include the overall patient health and reason for hospitalisation; the measurement and assessment of the outcome; and the variables associated with the environment that the subjects resided in. When interpreting the results of the three studies, it is therefore important to consider and acknowledge these variations in methodology as potential confounders. It is also important to acknowledge the differences in the methodologies of the three studies examined and what is feasible in the assessment of behaviour in a hospital setting compared to in a home environment.

### Statistical analysis

All three papers used appropriate statistical analysis tools to assess the results (Gilbert-Gregory et al., 2016; Gruen et al., 2014; and Gruen et al., 2017). Gilbert-Gregory et al. (2016) performed a Wilcoxon signed rank test to assess the data, which is appropriate for use in non-normally distributed data. The use of a Fisher Exact Chi-squared test was also appropriate for analysing categorical data. However, drawing strong conclusions from the results and the provided p-values is overly optimistic, as the differences in p-values were very slight and almost insignificant when evaluating the total stress-related behaviours (P <0.001 for treatment group, and P = 0.078 for control group) (Gilbert-Gregory et al., 2016). Gruen et al. (2017) did not use a transformation or test such as Kappa to reduce bias caused by the owner as the assessor. They also used a Wilcoxon test which was appropriate, however, the presentation and lack of disclosure of all p-values created confusion for the reader. Overall, however, the statistical analysis is appropriate for the data available (Gruen et al., 2017). Gruen et al. (2014) recognised that the data was ordinal and used the Cochran-Mantel-Haenszel test which was appropriate for the data. However, no information was provided as to the baseline scores that were used to compare to the final results, and so it is also confusing for the reader to determine the reliability of the results.

### Application

When evaluating the efficacy of trazodone in dogs who have undergone hospitalisation, it is important to acknowledge the limited available evidence on trazodone use in dogs, and the ongoing reliance on pharmacokinetic data from humans (Jay et al., 2013). It has been reported (Ciribassi & Ballantyne, 2014) that trazodone is helpful as an anxiolytic for events such as veterinary visits, separation anxiety and thunderstorms, for example, however, the onset of activity is generally delayed by up to an hour, and the effects induced by the drug can be unpredictable (Papich, 2016d). This is acknowledged by Gilbert-Gregory et al. (2016) through the assessor waiting at least 90 minutes before observing the study dogs to assess the effects of trazodone. Trazodone itself has a wide half-life range (166 ± 47 minutes) and in reaching peak plasma concentration (445 ± 271 minutes), therefore, it is important to consider this when adjusting the dosage to account for variations between individuals (Ciribassi & Ballantyne, 2014). Individual differences in baseline behaviour, personality characteristics, behavioural issues, confounding stimuli and use of concomitant medications all have the potential to influence the efficacy of trazodone use, and need to be considered when prescribing the drug as an anxiolytic for dogs experiencing stress induced by hospitalisation (Jay et al., 2013; Ciribassi & Ballantyne, 2014; and Gruen & Sherman, 2008).

### Safety and adverse effects

Trazodone hydrochloride has been administered to dogs orally without serious adverse effects reported (Beerda et al., 1998). At higher doses sedation commonly occurs, however this effect may be desirable (Beerda et al., 1998). Adverse events reported after oral administration included vomiting, gagging, colitis, increased excitement, sedation, increased appetite, and behavioural disinhibition such as getting onto counters (Gruen & Sherman, 2008), and hypersalivation (Jay et al., 2013). Intravenous administration of trazodone is not recommended (Beerda et al., 1998) as it appears to cause more serious adverse effects, with one study reporting that transient tachycardia and ataxia developed in all dogs involved in the study, and in another study 3/6 (50%) of the dogs briefly became aggressive (Jay et al., 2013). Caution should also be taken when administering trazodone concurrently with other drugs as it is highly metabolised by the cytochrome P450 enzymes, and these enzymes can be inhibited or induced if other drugs are administered alongside trazodone (Jay et al., 2013). Serotonin syndrome is also a possibility when using drugs that effect serotonin receptors, and although again the effects of this syndrome have not been observed or reported in dogs in trazodone studies, trazodone should be used with caution when concurrently administering drugs that act on the serotonergic system including selective serotonin reuptake inhibitors, tricyclic antidepressants, tramadol, and monoamine oxidase inhibitors (Lloyd, 2017). Overall, it is concluded that oral administration of trazodone hydrochloride within dosage limits (starting dose typically 2–5 mg/kg with maximum dose 19.5 mg/kg PO daily or 300 mg/dose in highly anxious or aggressive patients) appears to be safe for use in dogs (Lloyd, 2017; Jay et al., 2013; and Gruen & Sherman, 2008).

### Clinical application

The reduction of stress in canine patients undergoing hospitalisation not only improves the emotional welfare of these patients, but also results in improved health implications and reduced iatrogenic harm associated with veterinary care (Gilbert-Gregory et al., 2016). Incorporating an effective anxiolytic into a pre or postoperative protocol may assist veterinarians in reducing the effects of stress on their patients.

The goal of utilising trazodone in hospitalised dogs and dogs undergoing postsurgical confinement is to minimise the stress associated with the often necessary and common practice of hospitalisation and / or confinement – whether before or after surgery, before a noninvasive procedure such as a nail clip, or to allow for an aggressive or nervous dog to be more thoroughly examined without exacerbating the risk to a veterinarian, veterinary nurse, or owner. Other drugs such as acepromazine are not considered effective anxiolytics, and have even been shown to potentially increase anxiety and noise sensitivity depending on the dose used (Gilbert-Gregory et al., 2016; and Overall, 2013). There is, therefore, a need in the veterinary practice for an agent that also provides anxiolytic benefits. It is also important for veterinarians to administer a drug that is proven to reduce the physical and emotional consequences of stress if an agent is prescribed for that reason, otherwise the welfare of the dog may be at risk even if the administering veterinarian is satisfied with the act itself of prescribing a drug that will supposedly reduce the patients’ stress i.e., due to the placebo effect.

Trazodone is widely used in many clinics and is considered an effective means of reducing anxiety in their patients. Even in the analysed studies, the authors themselves were perceived to have a selection bias as they were expecting trazodone to be effective and produce positive outcomes in the study participants (Gruen et al., 2014; and Gruen et al., 2017). It is therefore important to consider the differences in the three studies evaluated when determining the effectiveness of trazodone in reducing signs of stress in hospitalised dogs and dogs confined postsurgery, as well as the rationale of trazodone use by veterinarians as determined by anecdotal and experiential evidence.

### Reflection

There is currently evidence of weak strength that demonstrates that trazodone reduces stress related behaviour in hospitalised dogs (Gilbert-Gregory et al., 2016), and weak evidence that it reduces stress related behaviour in dogs confined postsurgery (Gruen et al., 2014; and Gruen et al., 2017), however, it is difficult to compare trazodone to other anxiolytics due to the lack of studies into other medications and their efficacy in these contexts. Gilbert-Gregory et al. (2016) has the strongest evidence due to a better study design, however it also includes many confounding factors that weaken the evidence of trazodone efficacy such as the administration of concomitant medications, not utilising true controls, selection bias, and not accounting for the type of surgery and duration in hospital. Gruen et al. (2014; and 2017) demonstrates weak evidence reflecting the small sample sizes used in the studies, and the inconsistency of assessment of behaviour. Taken together the evidence of efficacy of trazodone in this context of postsurgical confinement is inadequate.

Given that all three studies are weakened by various limitations and confounding factors, further studies are required to provide conclusive evidence, utilising a larger sample size, standardised measurement and assessment tools strengthened by evidence surrounding dog behaviour such as the assessment tool utilised by Gilbert-Gregory et al. (2016) alongside a veterinary behaviourist, which was lacking in Gruen et al. (2014; and 2017), the use of healthy patients in a novel hospital environment when assessing hospitalised patients which would not be feasible in all studies, and incorporating blinding of administrators and assessors using a placebo and randomisation. Statistically, a sample size of 323 dogs would be required to detect a perfect change with 60–70% of dogs affected by the drug (Dhand & Khatkar, 2014). Also, in an ideal world, it would be important to ensure that trazodone is the sole therapeutic agent administered to subjects during the study to eliminate the confounding effects of other drugs. However, this would prove difficult unless a research population was utilised. Whilst there are practical constraints and financial limitations involved in acquiring strong evidence in this subject, the absence of evidence suggests that more research must be conducted in this field for appropriate and correct recommendations to be made about the use of trazodone within a veterinary clinic.

## Methodology

**Search strategy:** 

Databases searched and dates covered:	CAB Abstracts on OVID Platform 1973 to 2023 Week 4 PubMed 1920 – January 2023
Search strategy:	CAB Abstracts: (dog or dogs or canine or canines).mp. or exp dogs/ (trazodone or desyrel or oleptro).mp. (stress or stressed or stressful or anxiety or anxious or nervous or nervousness or distressed or distress or fear or fearful).mp. (hospital or hospitals or hospitalised or hospitalized or clinic or clinics or consult or consultation or surgery).mp. 1 and 2 and 3 and 4 PubMed: dog or canine trazodone or desyrel or oleptro stress or stressed or stressful or anxiety or anxious or nervous or nervousness or distressed or distress or fear or fearful hospital or hospitalised or hospitalized or clinic or consult or consultation or surgery 1 and 2 and 3 and 4
Dates searches performed:	01 Feb 2023

**Exclusion / inclusion criteria:** 

Exclusion:	Not related to the PICO parameters (non-dog patients; not evaluating the effects of administration of trazodone; not evaluating effect of administration of trazodone for a hospital setting or postsurgical confinement). Case studies. Systematic reviews. Study summary / article.
Inclusion:	Dog subjects. Evaluating effects of trazodone in a hospital setting (in hospital; confined after hospital).

**Search outcome:** 

Database	Number of results	Excluded – Case study	Excluded – Non-canine	Excluded – Not evaluating effects	Excluded – Not evaluating in hospital setting or confinement	Excluded – Article	Excluded – Systematic review	Total relevant papers
CAB Abstracts	14	4	0	1	3	3	1	2
PubMed	18	4	2	2	4	3	1	2
Total relevant papers when duplicates removed								3

## ORCiD

Lara Dillon: https://orcid.org/0000-0001-5843-3421

## Conflict of interest

The author declares no conflicts of interest.
